# In-vivo high resolution imaging of optic nerve head drusen using spectral-domain Optical Coherence Tomography

**DOI:** 10.1186/1471-2342-10-11

**Published:** 2010-06-07

**Authors:** Ravi K Murthy, Laura Storm, Sandeep Grover, Vikram S Brar, Kakarla V Chalam

**Affiliations:** 1 Department of Ophthalmology, College of Medicine, University of Florida, Jacksonville, Florida, USA

## Abstract

**Background:**

Optic nerve head drusen (ONHD) are white calcareous deposits, seen either superficially on the optic nerve head or buried within it. Diagnosis of ONHD is made by one or more ways: clinical exam, autofluorescence, ultrasound of the optic nerve, CT scan and/or visual field examination. The present study describes features of ONHD based on another diagnostic modality, the spectral-domain OCT (Spectralis).

**Methods:**

This is a retrospective case series of 5 patients with bilateral ONHD with a best-corrected visual acuity of 20/20 and no other posterior segment pathology. All the patients underwent fundus photography, fundus autofluorescence, B-scan ultrasonography, Spectralis OCT and Humphrey 30-2 threshold visual fields.

**Results:**

All 5 patients had surface ONHD which were autofluorescent and echodense on B-scan ultrasonography. Spectralis OCT findings in the corresponding areas include 'scattered spots with high reflectivity' casting a shadow underneath. The reflectivity can be distinctly differentiated from the blood vessels on the optic nerve. Two patients had an arcuate scotoma on the Humphrey visual fields. No correlation was found between the changes on Spectralis OCT with that of visual field.

**Conclusions:**

Spectralis OCT is another useful ancillary investigation in the diagnosis of ONHD and we describe the features in the present study.

## Background

Optic Nerve Head Drusen (ONHD) are white calcareous deposits that are generally asymptomatic and are bilateral in 66-85% of cases [1]. They are characteristically found in the pre-laminar region of the ONH and are believed to arise from the debris due to long standing axonal stasis in the nerve fibre layer [2]. Histopathologically, the drusen contain multiple deposits of calcium crystals that vary in size (5 to 1000 microns) [2,3]. These crystals mechanically compress the overlying nerve fibre layer resulting in progressive visual field defects [4].

The diagnosis of ONHD is based upon a strong index of suspicion and confirmed by B-scan ultrasound of the posterior segment, which shows echodense structure with acoustic shadowing. CT scan may be required in the rare case when ultrasound is inconclusive [1-3]. Time-domain Stratus OCT has been used in recent years to document the nerve fibre layer changes associated with drusen [5]. However, the poor resolution of time-domain OCT prevents the visualization of changes seen as a result of mechanical compression due to the drusen.

Spectral-domain Optical Coherence Tomography (SD-OCT) by its ability to acquire 40000 A-scans per second provides high resolution images of the Optic nerve head [6]. In this case series we describe SD-OCT findings in 5 cases of ONHD.

## Methods

This is a retrospective case series report of 5 patients with ONHD in whom changes seen on spectral domain OCT (SD-OCT) were documented. Approval for this study was obtained from the University of Florida Institutional Review Board-03 (Reference number: 2008-003). Five patients with bilateral ONHD with best corrected visual acuity 20/20 and no other posterior segment pathology were included in this series. All the patients underwent a detailed ocular examination, posterior segment digital fundus photography (Zeiss FF450, Carl Zeiss, US), fundus angiography to document autofluorescence (Spectralis, Heidelberg Engineering, Germany) and B-scan ultrasonography of the posterior segment (Sonomed Inc, US).

Optic nerve head scans, using the Spectralis OCT (Heidelberg Engineering, Germany) were done on all the patients by an experienced operator using the raster lines protocol. The area scanned was 4 mm by 4 mm at 50 micron intervals. Cross-sectional images, one per second, were obtained with a longitudinal resolution of 7 μm. Patient's fixation was monitored by asking the patient to look at an internal fixation or by guiding through an infrared monitoring camera.

## Results and Discussion

**Figure 1:**
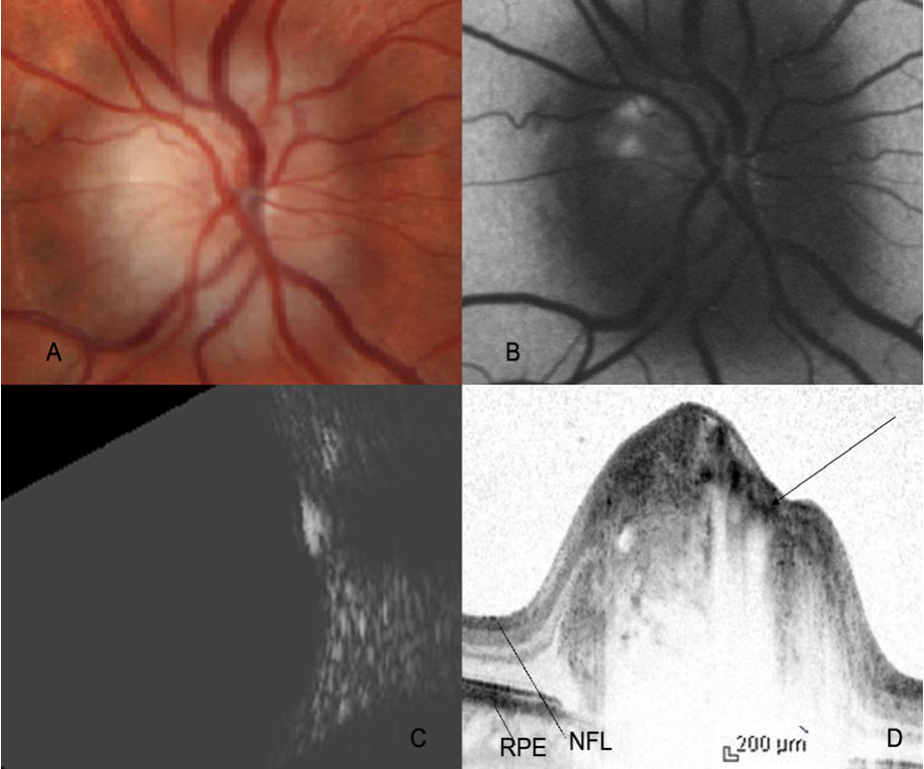
**Color fundus photograph, FAF imaging, B-scan ultrasound and Spectralis OCT of subject 1**. Color fundus photograph revealed yellow white surface calcareous deposits on the optic nerve head (A). FAF imaging of the same subject demonstrated autofluorescence of the surface drusen (B). B-Scan ultrasound showed echodense lesion overlapping the optic nerve shadow. The reflectivity persisted at 30 decibels (C). Cross-sectional Spectralis OCT image of drusen revealed the 'cap' sign (arrow), an empty space lined by high reflectivity signal (D). NFL: Nerve fibre layer, RPE: Retinal pigment epithelium.

**Figure 2:**
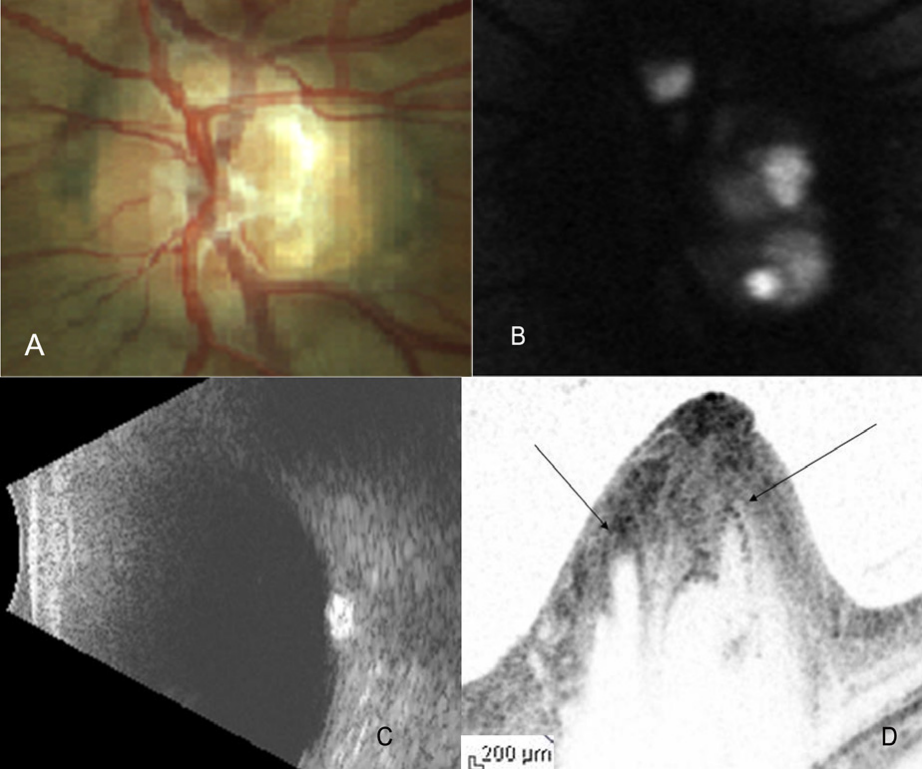
**Color fundus photograph (A), FAF image (B), B-Scan ultrasound (C) and cross-sectional Spectralis OCT image (D) of subject 2 in the study**. The intensity of FAF image has been turned down to highlight the high reflectivity of the drusen. Arrows on the Spectralis OCT cross-sectional image indicate the position of the drusen.

**Figure 3:**
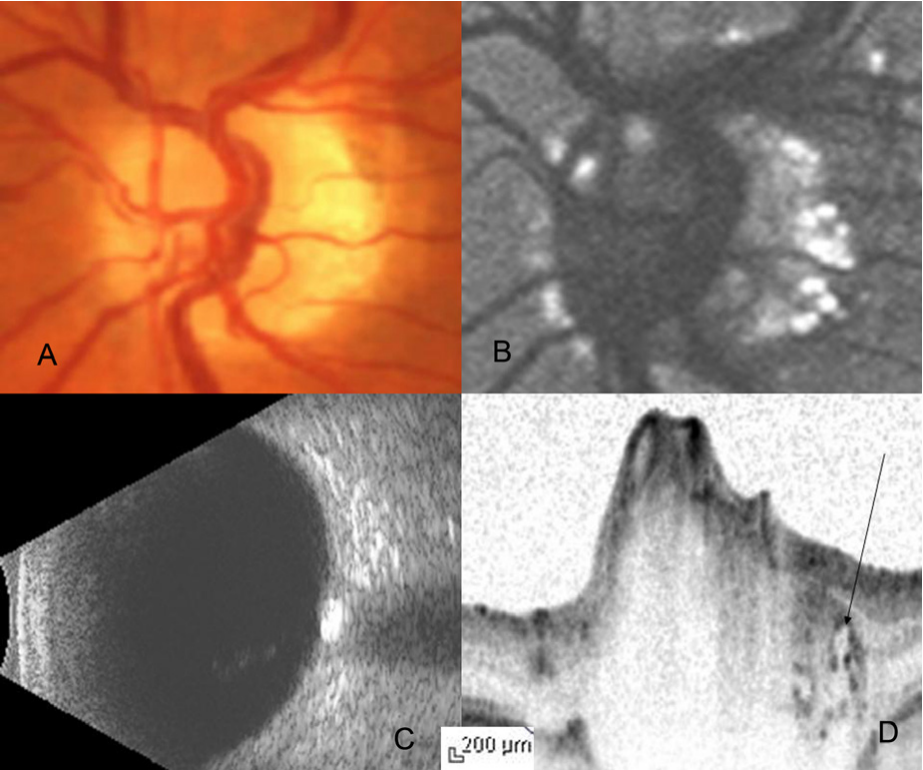
**Color fundus photograph (A), FAF image (B), B-Scan ultrasound (C) and cross-sectional Spectralis OCT image (D) of subject 3 in the study**. The drusen are located along the margin of the optic nerve head. Arrow indicates the corresponding location of the drusen on the Spectralis OCT cross-sectional image.

The median age of the patients was 51 years (range 17-53 years). The optic nerve head of all the patients showed glistening surface deposits characteristic of drusen (Figure 1A, 2A, 3A), which demonstrated fundus autofluorescence (Figure 1B, 2B, 3B). Further, the diagnosis of ONHD was confirmed by the echodense appearance on B-Scan ultrasonography (Figure 1C, 2C, 3C). In the areas on the ONH which were autofluorescent due to the drusen, imaging with the Spectralis OCT revealed cluster of deposits with high reflectivity, corresponding to the location of the drusen. Some of these deposits cast a shadow underneath and had lacunae like appearance (Figure 1D, 2D, 3D). Histopathologically, the drusen contain multiple deposits of calcium crystals that vary in size from 5 to 1000 microns [2]. The deposition is seen within the mitochondria of the intact axons. With the disruption of axons, the nidus of calcification expands in the extracellular space with subsequent coalescence of adjacent foci to form drusen [3].

SD-OCT by its ability to acquire 100 times more number of A-scans as compared to Stratus OCT provides high resolution images of the cross section of the optic nerve head, a close approximation to the histopathological section [6-9]. In our case, the SD-OCT showed dense spots within the nerve fibre layer of the ONH causing shadowing underneath, corresponding to the calcium content of the drusen. In areas corresponding to the drusen, lacunar areas with a single spot of high reflectivity was noted, which we describe as a 'cap' sign. These probably correspond to the nidus of calcification seen histopathologically in the early stages of the formation of drusen. This feature helps in differentiating circular areas of decreased reflectivity seen as the major blood vessels cast an optical shadow underneath. In addition, SD-OCT is useful in detecting the buried drusen which are not obvious on clinical examination.

Visual field changes described in cases of ONHD are characteristically arcuate scotomas corresponding to the nerve fibre layer defects arising out of mechanical compression due to the drusen [10]. In our series, two patients had an arcuate scotoma on the Humphrey visual fields. However, these changes could not be correlated as there were no corresponding baseline scans available. These changes are also commonly seen in surface rather than buried drusen which was the case in two of our patients. Further study of a larger population of patients with serial follow up is required to validate the use of spectral-domain OCT in the management of patients with ONHD.

## Conclusions

In summary, SD-OCT is a useful ancillary investigation in the diagnosis of optic nerve head drusen and in documenting the changes seen in retinal nerve fibre layer due to the compressive effects of the drusen.

## List of abbreviations

ONHD: Optic Nerve Head Drusen; SD-OCT: Spectral-domain Optical Coherence Tomography.

## Competing interests

The authors declare that they have no competing interests.

## Authors' contributions

RK, SG, KVC were involved in the conception and design of the study; RKM, LS was involved in the acquisition of data; RKM, SG, KVC and VSB were involved in the analysis of the data and preparation of the manuscript.

All authors have read and approved the final manuscript.

## Pre-publication history

The pre-publication history for this paper can be accessed here:

http://www.biomedcentral.com/1471-2342/10/11/prepub
